# Supplementary material for: Room temperature organic magnets derived from sp3 functionalized graphene
Source: Nat Commun. 2017 Feb 20;8:14525. doi: 10.1038/ncomms14525 (PMC5321725; doi:10.1038/ncomms14525)
Supplement: Supplementary Information — Supplementary Figures and Supplementary Tables [file ncomms14525-s1.pdf]

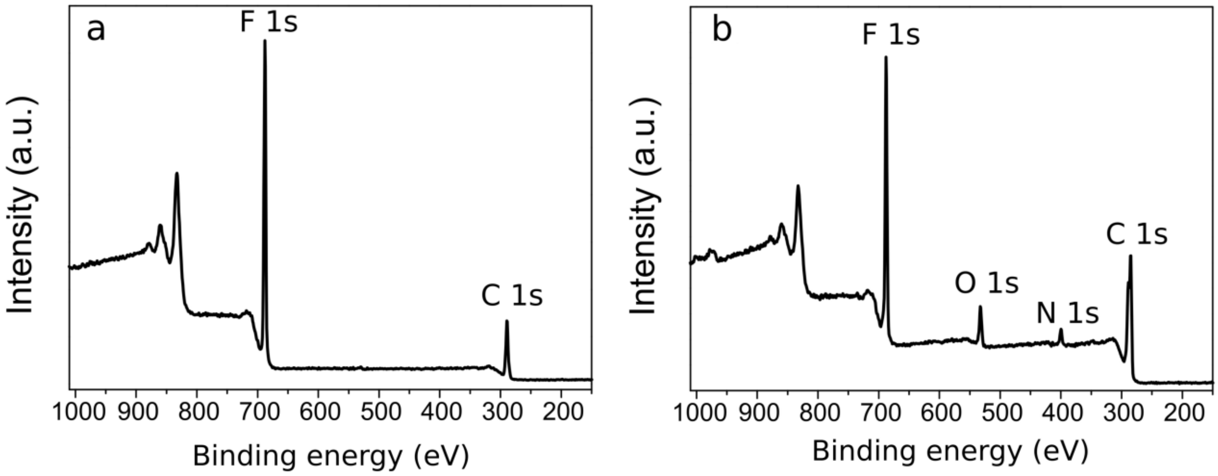

Supplementary Figure 1. X-ray photoelectron spectroscopy (XPS) patterns. (**a**) Survey XPS spectrum of fluorographene (GF). (**b**) Survey XPS spectrum of hydroxofluorographene (G(OH)F).

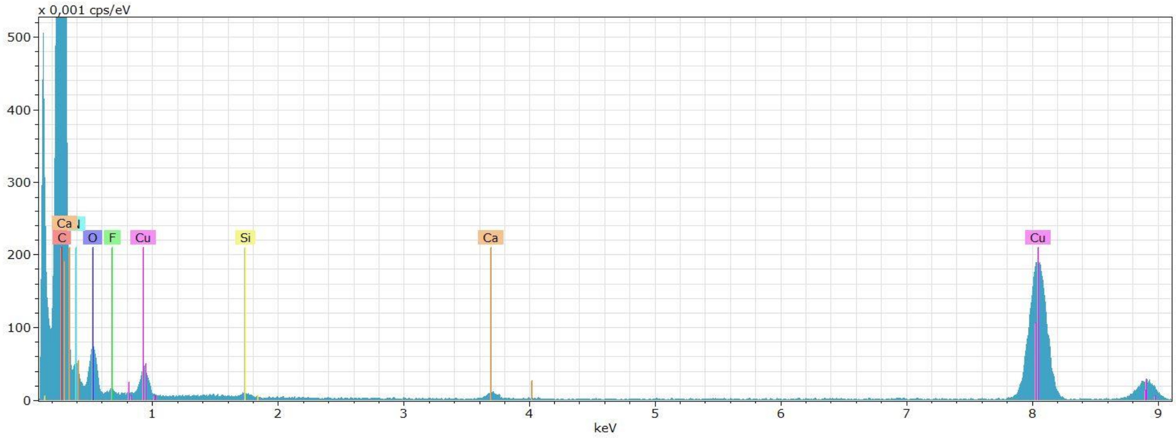

Supplementary Figure 2. The energy-dispersive X-ray (EDX) spectrum of the G(OH)F sample obtained from high-resolution transmission electron microscopy (HRTEM) imaging. The higher amount of carbon and cooper in the spectrum is caused by the TEM grid. The calcium and silicon may origin from the manipulation with the TEM grid but also from the glass vials where the sample was stored.

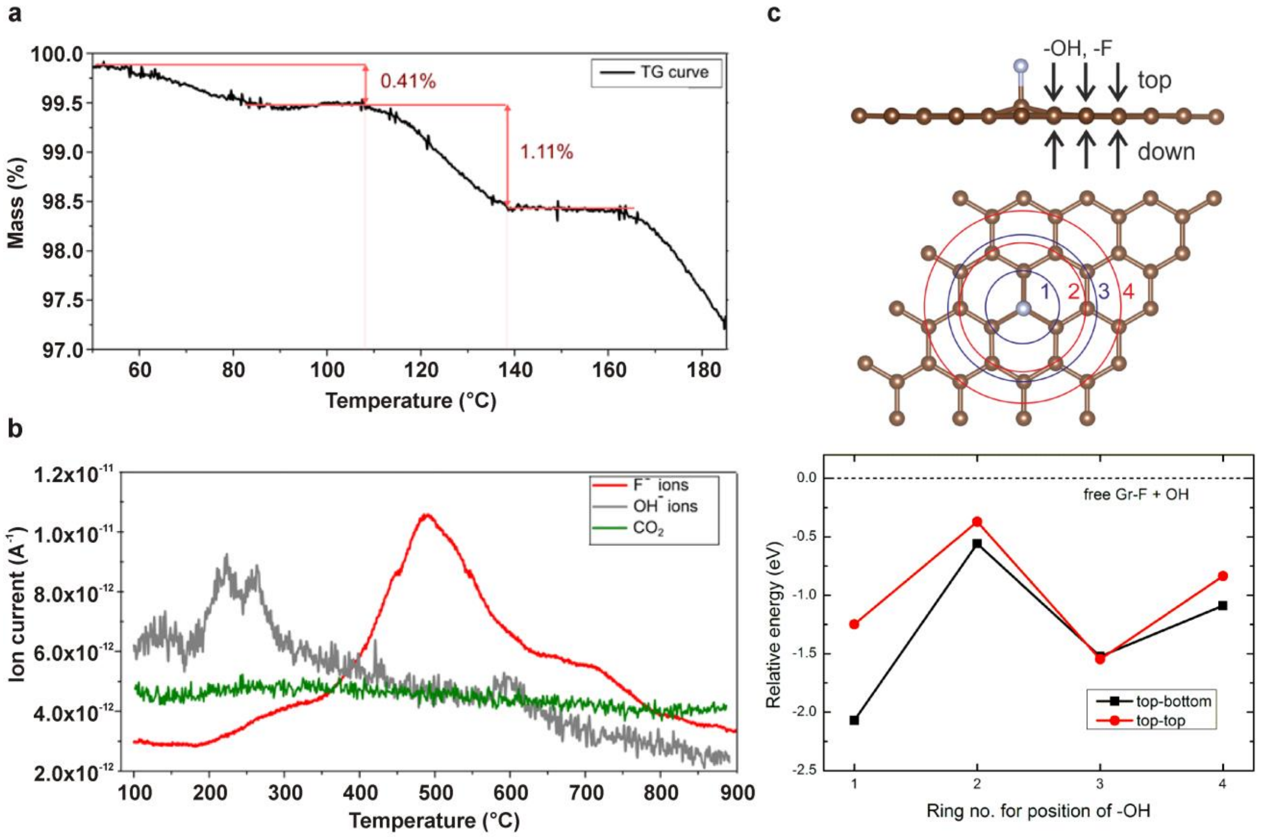

Supplementary Figure 3. Thermogravimetric (TG) and evolved gas analysis (EGA) measurements and theoretical evaluation of the stability of the –OH group on various adsorption sites. (**a**) TG curve of the G(OH)F sample. (**b**) EGA measurements of the G(OH)F sample with continuous detection of released ions and gases. (**c**) Theoretical evaluation of the stability of the –OH group on various adsorption sites (side and top views) in the presence of –F on graphene adopting the identical strategy published elsewhere. The ring #1 represents the nearest neighbors, ring #2 the second nearest neighbors, etc.) The relative energy of –OH group is plotted for different positions (on the same side in red, on the opposite side in black) with respect to free G – F + OH. The plot documents that the stability of G-OH bond depends on the local environment, i.e., presence and distance of fluorine atoms, which may explain the two peaks for the –OH groups in EGA of G(OH)F.

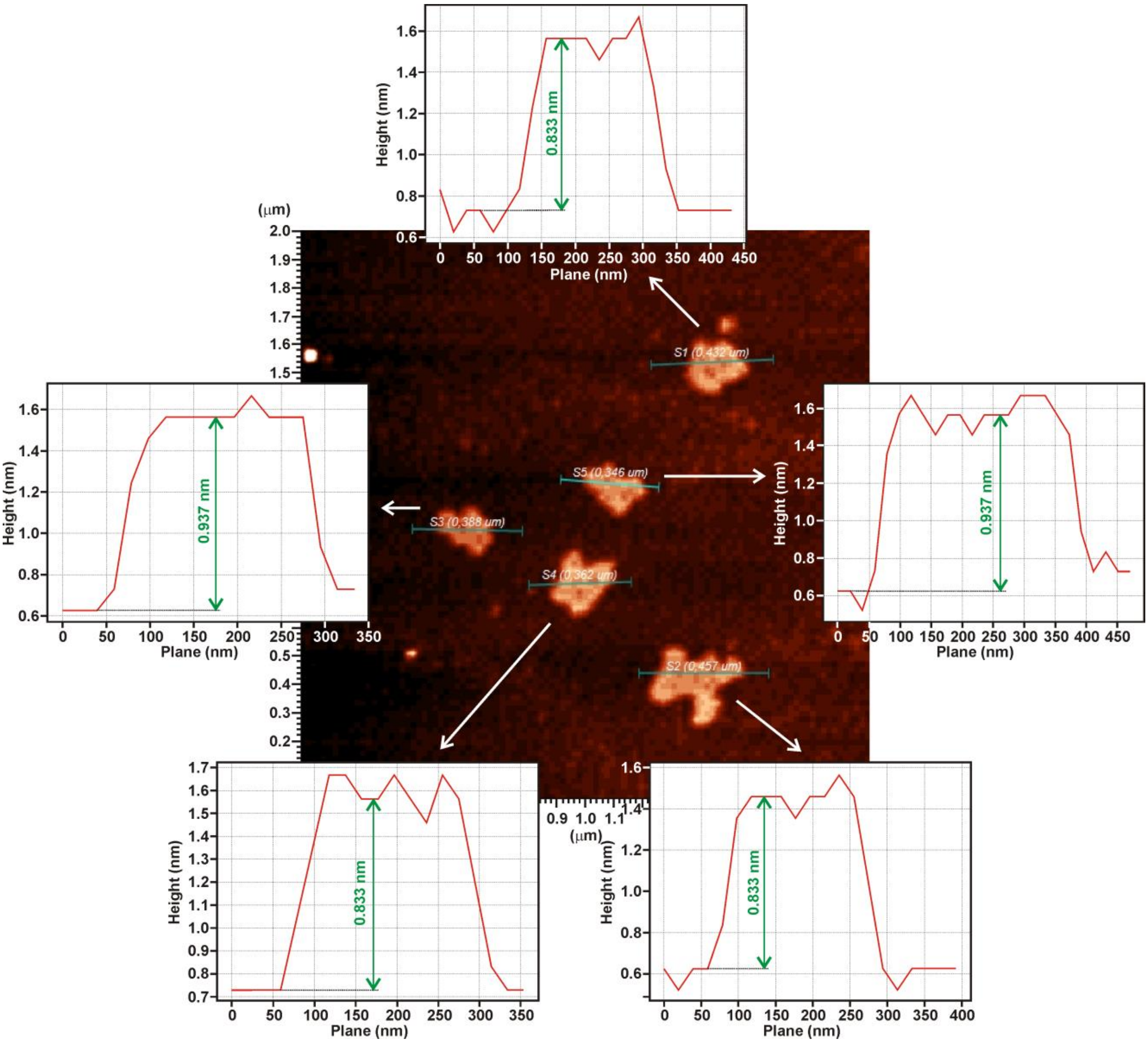

Supplementary Figure 4. Atomic force microscopy (AFM) measurements. Representative AFM image of G(OH)F with the height profiles demonstrating the sample's single-sheet character.

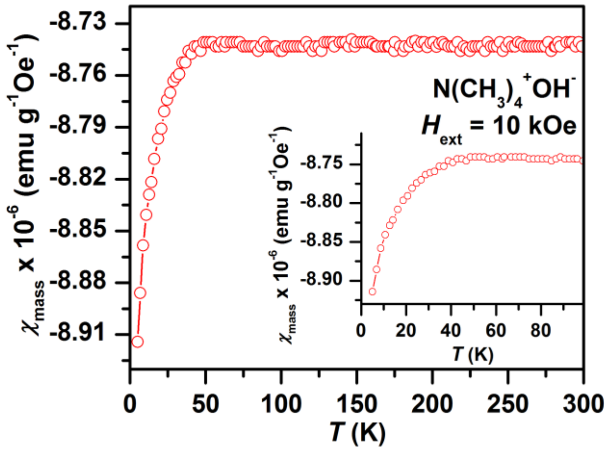

Supplementary Figure 5. Temperature evolution of the mass susceptibility (χ_{mass}) of tetramethylammonium hydroxide. The value of χ_{mass} over the measured temperature range was near-constant, which is typical for diamagnetic materials.

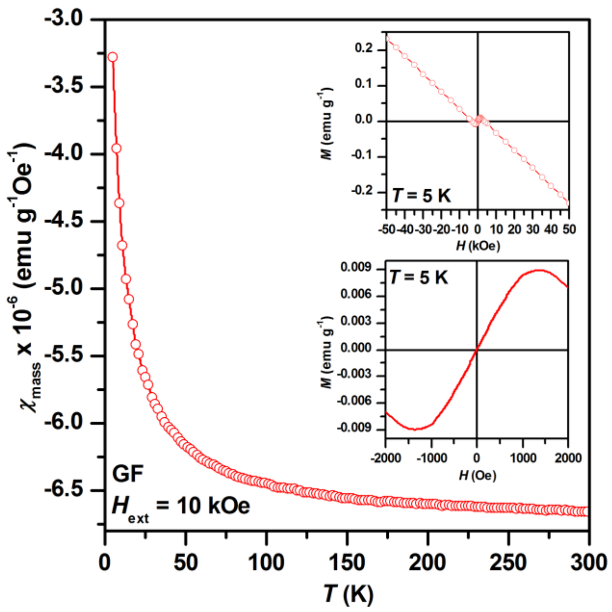

Supplementary Figure 6. Temperature evolution of the mass magnetic susceptibility (χ_{mass}) of GF, measured under an external magnetic field of 10 kOe. The insets show the behavior of the hysteresis loops of GF, recorded at a temperature of 5 K.

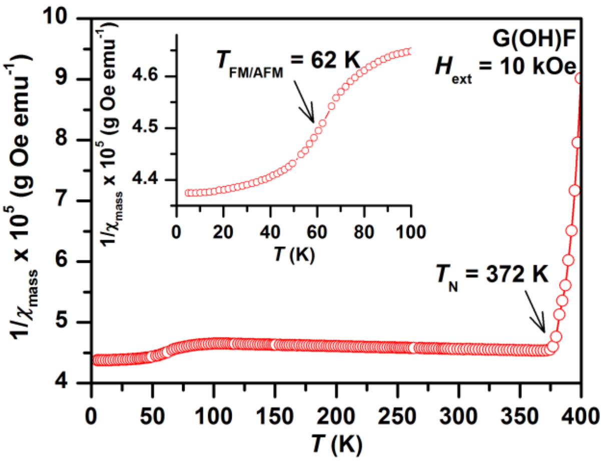

Supplementary Figure 7. Temperature evolution of the mass magnetic susceptibility (χ_{mass}) of G(OH)F, measured under an external magnetic field of 10 kOe. Reciprocal χ_{mass} vs. temperature curve with arrows indicating the passage from the paramagnetic to antiferromagnetic state (T_N, the Néel temperature) and passage from antiferromagnetic to ferromagnetic regime ($T_{FM/AFM}$, see the inset). Note: The paramagnetic signal from non-interacting centres was subtracted from $1/\chi_{mass}$ data.

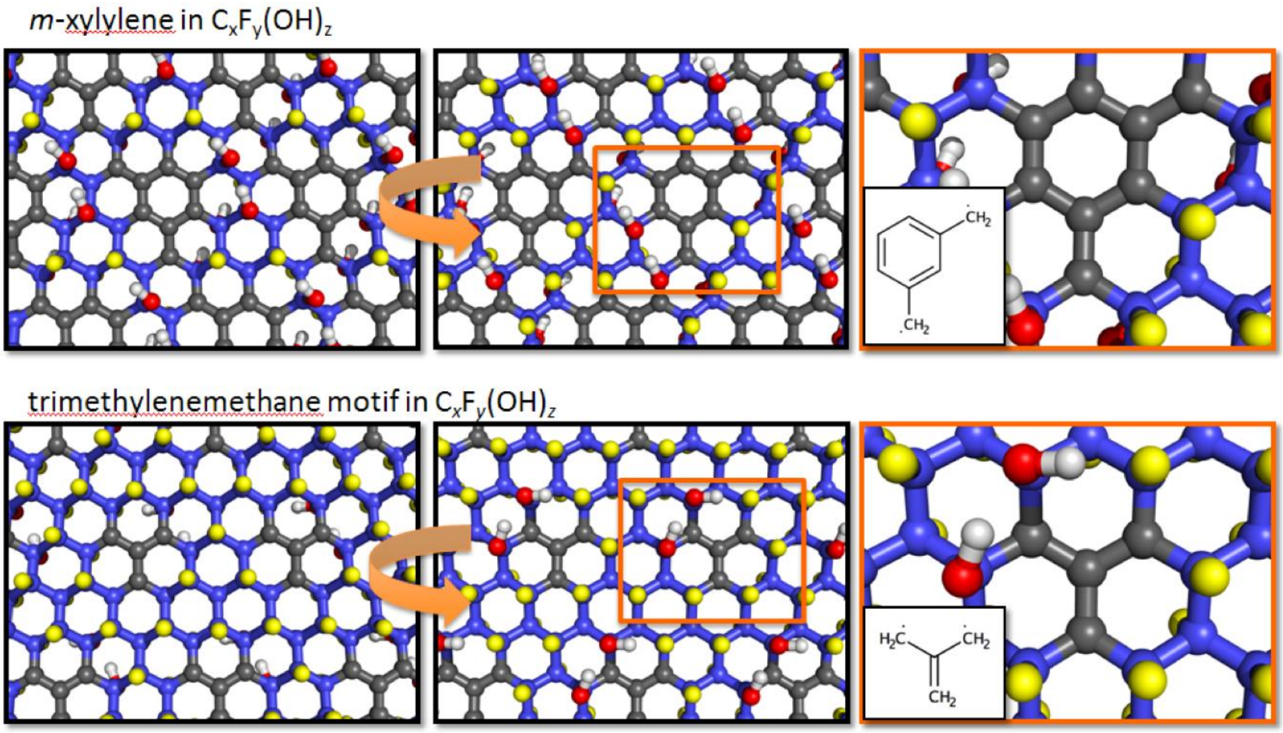

Supplementary Figure 8. Models of diradical motifs in the G(OH)F system. (**a**) *m*-xylylene motif in $C_xF_y(OH)_z$. (**b**) Trimethylenemethane motif in $C_xF_y(OH)_z$. The left and middle panels show the diradical motifs from above and below the graphene plane, while the right panels show the details of the particular diradical motif. The insets in the right panels show the structures of the two diradical motifs. Fluorine atoms are shown in yellow, hydrogen atoms in white, oxygen atoms in red, sp^3 carbon atoms in blue, and sp^2 carbon atoms in grey.

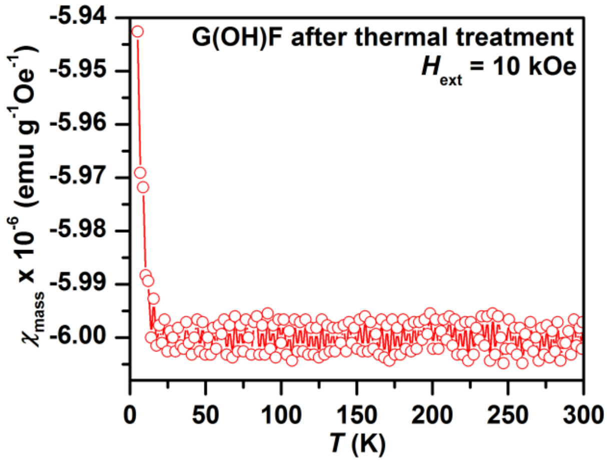

Supplementary Figure 9. Thermal evolution of the mass magnetization (χ_{mass}) of the G(OH)F sample after thermal treatment at 200 °C. The negative χ_{mass} values over the whole temperature interval imply diamagnetic behavior without any sign of magnetically ordered (ferromagnetic or antiferromagnetic) state.

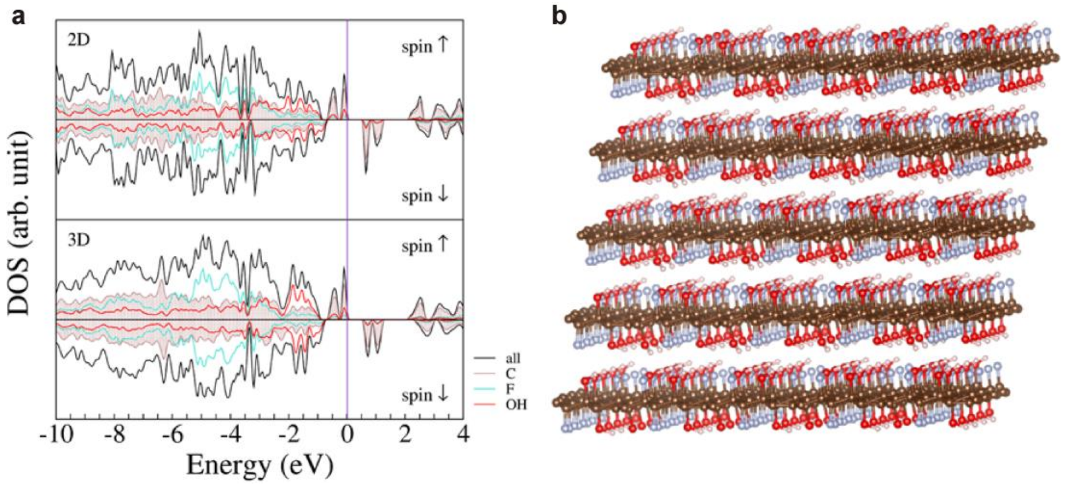

Supplementary Figure 10. **Computational modelling of the effect of 3D stacking of G(OH)F sheets on its magnetic features.** (**a**) Density of states (DOS) for monolayer (2D) G(OH)F and a hypothetical 3D bulk G(OH)F. (**b**) Structure of 3D bulk G(OH)F.

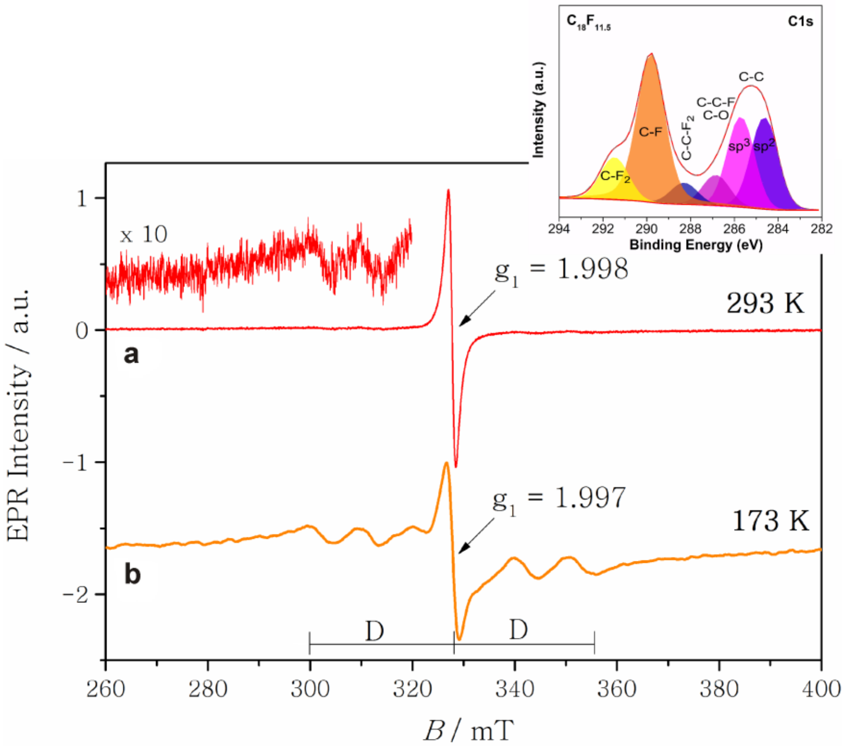

Supplementary Figure 11. EPR measurements. (**a**) X-band EPR spectrum of the $C_{18}F_{11.5}$ sample recorded at 293 K. (**b**) X-band EPR spectrum of the $C_{18}F_{11.5}$ sample recorded at 173 K. The low-field region of the spectrum has been magnified (x 10, upper trace) to more clearly show the presence of triplet species coexisting with the doublet species. The EPR spectra are averaged and accumulated composites of the two scans. The inset shows the high-resolution C 1s XPS pattern providing quantification of the $sp^3/(sp^2 + sp^3)$ ratio (0.735) for the $C_{18}F_{11.5}$ sample.

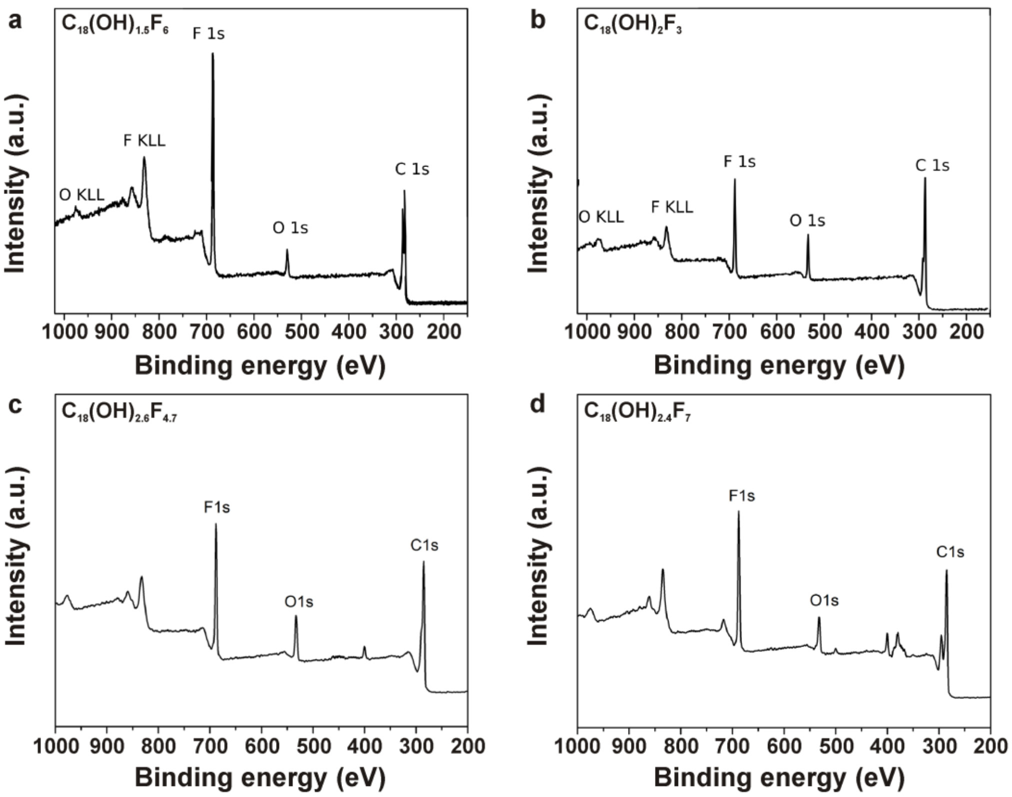

Supplementary Figure 12. Survey XPS patterns of other hydroxofluorographenes, $C_{18}(OH)_{1.5}F_6$, $C_{18}(OH)_2F_3$, $C_{18}(OH)_{2.6}F_{4.7}$, and $C_{18}(OH)_{2.4}F_7$. (**a**) Survey XPS pattern of hydroxofluorographene with a stoichiometry of $C_{18}(OH)_{1.5}F_6$ prepared from the $CF_{0.8}$ precursor. (**b**) Survey XPS pattern of $C_{18}(OH)_2F_3$ prepared from the $CF_{0.55}$ precursor. (**c**) Survey XPS pattern of $C_{18}(OH)_{2.6}F_{4.7}$ prepared through H_2O_2. (**d**) XPS pattern of $C_{18}(OH)_{2.4}F_7$ prepared through tert-BuOOH.

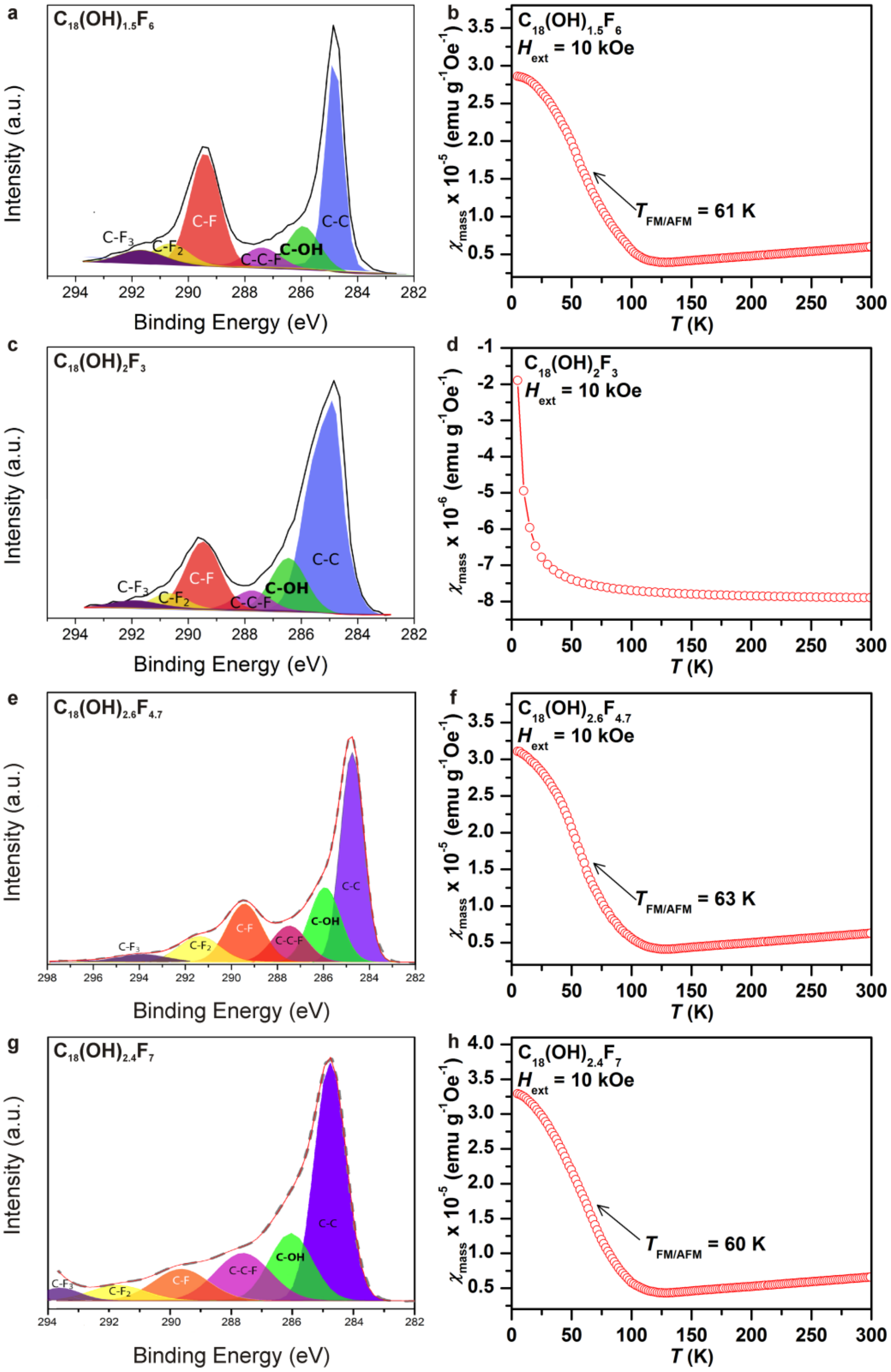

Supplementary Figure 13. XPS and magnetic characterization of other hydroxofluorographenes, $C_{18}(OH)_{1.5}F_6$, $C_{18}(OH)_2F_3$, $C_{18}(OH)_{2.6}F_{4.7}$, and $C_{18}(OH)_{2.4}F_7$. (**a**) High resolution C 1s XPS spectrum of $C_{18}(OH)_{1.5}F_6$, prepared from the $CF_{0.8}$ precursor. (**b**) Temperature profile of the mass susceptibility (χ_{mass}) of $C_{18}(OH)_{1.5}F_6$ between 5 and 300 K in an external magnetic field of 10 kOe. (**c**) High resolution C 1s XPS spectrum of $C_{18}(OH)_2F_3$, prepared from the

$CF_{0.55}$ precursor. (**d**) Temperature profile of χ_{mass} of $C_{18}(OH)_2F_3$ between 5 and 300 K in an external magnetic field of 10 kOe. (**e**) High resolution C 1s XPS spectrum of $C_{18}(OH)_{2.6}F_{4.7}$, prepared through H_2O_2. (**f**) Temperature profile of χ_{mass} of $C_{18}(OH)_{2.6}F_{4.7}$ between 5 and 300 K in an external magnetic field of 10 kOe. (**g**) High resolution C 1s XPS spectrum of $C_{18}(OH)_{2.4}F_7$, prepared through tert-BuOOH. (**h**) Temperature profile of χ_{mass} of $C_{18}(OH)_{2.4}F_7$ between 5 and 300 K in an external magnetic field of 10 kOe. Note: The paramagnetic signal from non-interacting centres was subtracted from all χ_{mass} data for $C_{18}(OH)_{1.5}F_6$, $C_{18}(OH)_{2.6}F_{4.7}$, and $C_{18}(OH)_{2.4}F_7$ sample.

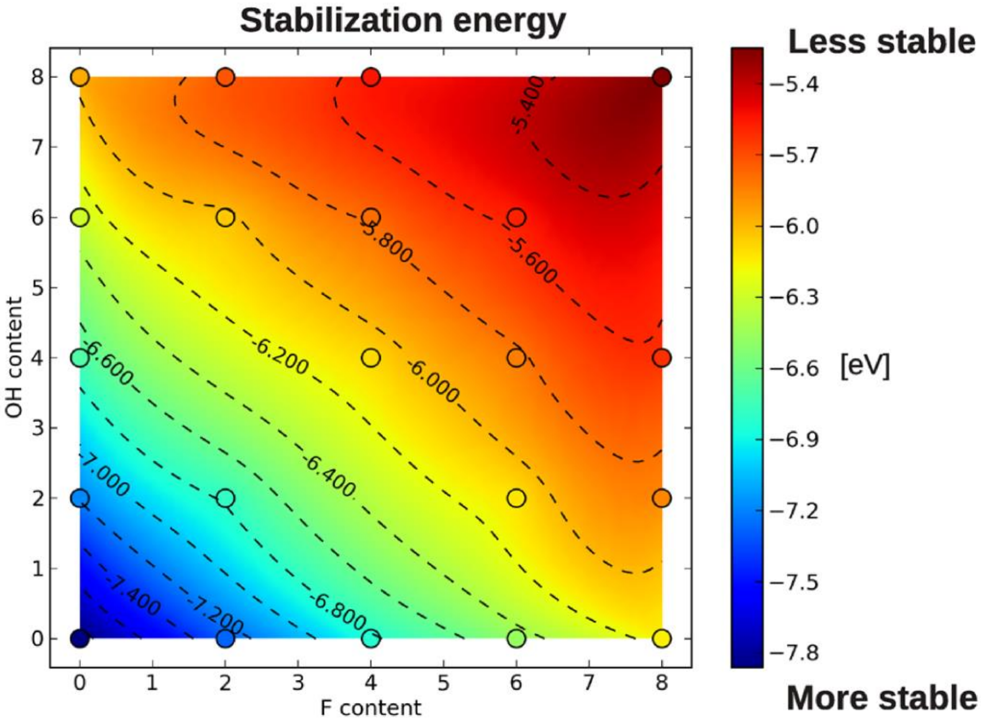

Supplementary Figure 14. Thermodynamic stability. The mean stabilization energies of $C_{18}(OH)_yF_x$ systems as a function of their stoichiometry. Each point represents an ensemble average over 32 low-energy structures selected from 64 random samples.

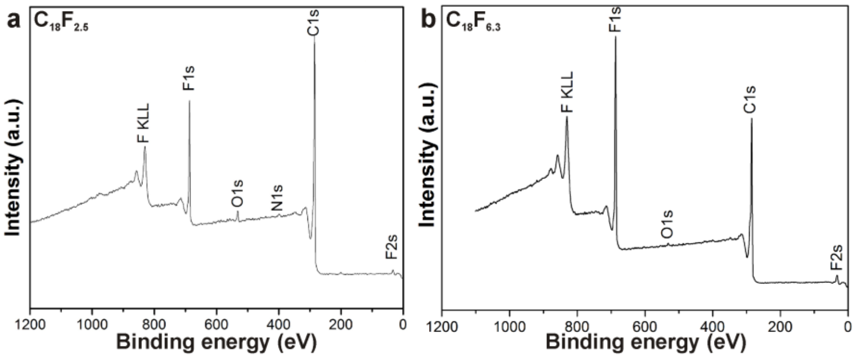

Supplementary Figure 15. Survey XPS patterns of two partially defluorinated fluorographenes, $C_{18}F_{2.5}$ and $C_{18}F_{6.3}$. (**a**) Survey XPS pattern of partially defluorinated fluorographene with a stoichiometry of $C_{18}F_{2.5}$, prepared from C_1F_1 by a controlled thermal treatment (for details, see the Methods section). (**b**) Survey XPS pattern of partially defluorinated fluorographene with a stoichiometry of $C_{18}F_{6.3}$, prepared from C_1F_1 by a controlled thermal treatment (for details, see the Methods section).

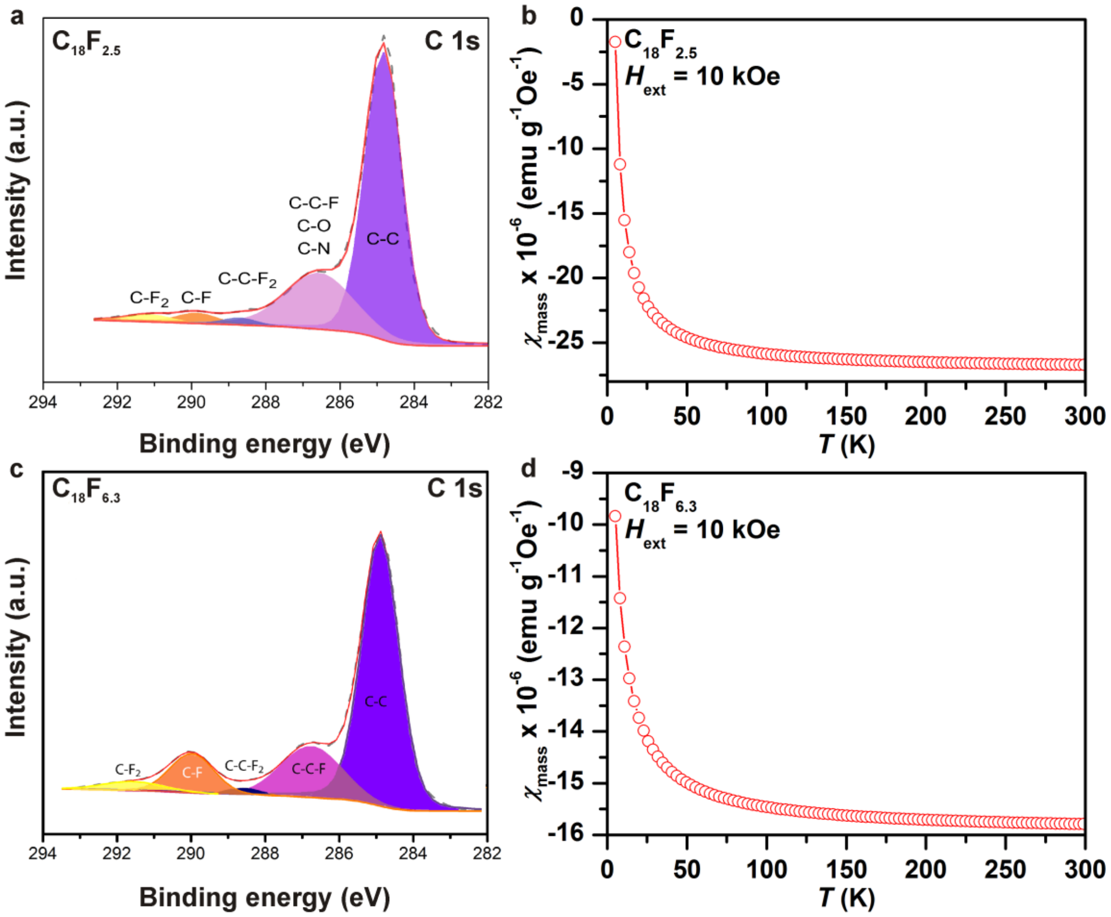

Supplementary Figure 16. XPS measurements and magnetic characterization of the two partially defluorinated fluorographenes, $C_{18}F_{2.5}$ and $C_{18}F_{6.3}$. (**a**) High-resolution C 1s XPS spectrum of $C_{18}F_{2.5}$, prepared by a controlled thermal treatment of the C_1F_1 precursor. (**b**) Temperature profile of the mass susceptibility (χ_{mass}) of $C_{18}F_{2.5}$ between 5 and 300 K in an external magnetic field of 10 kOe. (**c**) High-resolution C 1s XPS spectrum of $C_{18}F_{6.3}$, prepared by a controlled thermal treatment of the C_1F_1 precursor. (**d**) Temperature profile of χ_{mass} of $C_{18}F_{6.3}$ between 5 and 300 K in an external magnetic field of 10 kOe.

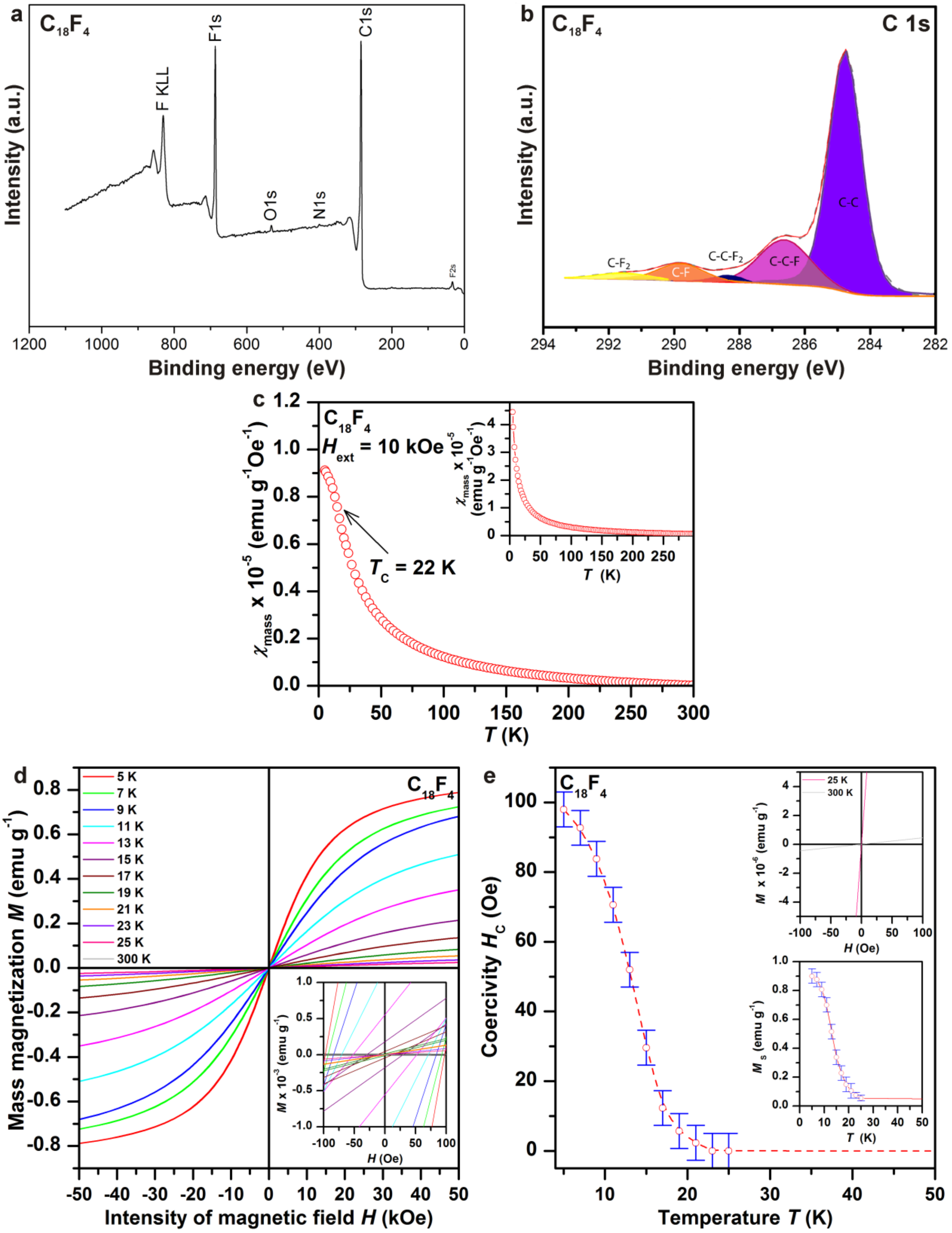

Supplementary Figure 17. XPS measurements and magnetic characterization of partially defluorinated fluorographene, $C_{18}F_4$. (**a**) Survey XPS pattern of $C_{18}F_4$, prepared by a controlled thermal treatment of the $C_1F_{1.1}$ precursor (for details, see Methods section). (**b**) High-resolution C 1s XPS spectrum of $C_{18}F_4$. (**c**) Temperature profile of the ferromagnetic mass susceptibility (χ_{mass})

of $C_{18}F_4$ between 5 and 300 K in an external magnetic field of 10 kOe showing a transition from the ferromagnetic to paramagnetic state at a Curie temperature of 22 K. The inset shows the temperature evolution of χ_{mass} with both ferromagnetic and paramagnetic contributions; the purely paramagnetic signal comes from the isolated paramagnetic centers, which are mostly located at the edges of the $C_{18}F_4$ sheets. (**d**) Isothermal magnetization curves of $C_{18}F_4$ at temperatures ranging from 5 to 300 K. The insets show the profile of the material's hysteresis loops around the origin, demonstrating its hysteretic behavior below 22 K, under which conditions it exists in the ferromagnetic state. (**e**) The temperature dependence of the coercivity (H_C) of $C_{18}F_4$. The insets show the profile of the hysteresis loop measured at temperatures of 25 and 300 K, and the temperature evolution of the saturation magnetization (M_S) derived from the extrapolation of the respective hysteresis loops.

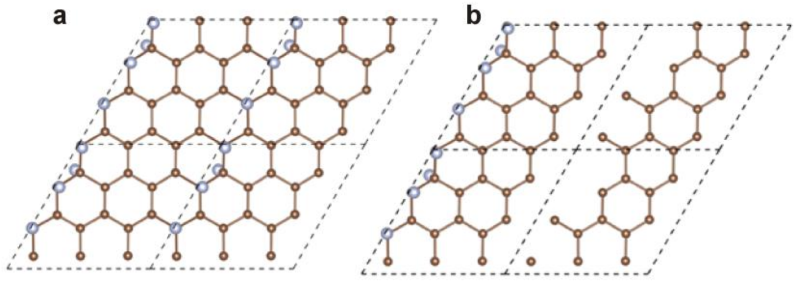

Supplementary Figure 18. Structure of $C_{18}F_4$ which can be considered as an *sp*^{3} structural defect in the *sp*^{2} graphene lattice. (**a**) The *sp*^{3} carbon atoms make a cut along zig-zag line and (**b**) virtually generate graphene ribbon with zig-zag edge (*sp*^{3} carbons and fluorine adatoms are hidden). The carbon atoms are shown in brown and fluorine atoms in cyan.

Supplementary Table 1. Concentrations of selected metals in the G(OH)F sample, as determined by ICP-MS.

Sample	Ti (ppm)	Cr (ppm)	Fe (ppm)	Co (ppm)	Ni (ppm)	Cu (ppm)	Zn (ppm)
G(OH)F	0.31	3.8	2.38	0.2	<0.00	0.68	23.27

Supplementary Table 2. Abundances of ferromagnetic (FM) and antiferromagnetic (AFM) ground states (%) of $C_{18}(OH)_yF_x$.

x	*y*	% of FM ground states	% of AFM ground states
0	0	0.0	0.0
0	2	0.0	0.0
0	4	6.3	0.0
0	6	15.6	0.0
0	8	18.8	53.1
2	0	0.0	0.0
2	2	0.0	0.1
2	6	6.3	9.4
2	8	12.5	62.5
4	0	21.9	0.0
4	4	6.3	3.1
4	6	12.5	31.3
4	8	12.5	53.1
6	0	9.4	9.4
6	2	18.8	15.6
6	4	28.1	21.9
6	6	3.1	59.4
8	0	12.5	40.6
8	2	12.5	46.9
8	4	12.5	50.0
8	8	15.6	25.0
